# *Terminalia buceras* (Syn. *Bucida buceras*) and Retinal Oxidative Stress: A Critical Review of Published Evidence and Its Relevance to Dry Age-Related Macular Degeneration

**DOI:** 10.3390/ijms27209267

**Published:** 2026-10-19

**Authors:** Lidianys María Lewis-Luján, Annette Pulcherie Iloki-Lewis, Diego Emmanuel Guerrero-Magaña, Juan Carlos Galvez-Ruiz, Cinthia Jhovanna Perez-Martinez, Judas Tadeo Vargas-Durazo, Sergio Trujillo-López, Simon Bernard Iloki-Assanga

**Affiliations:** 1 Department of Biological Chemical Sciences, University of Sonora, Luis Encinas y Rosales, Centro, Hermosillo 83000, Sonora, Mexico; lidianys.lewis@unison.mx (L.M.L.-L.); diego.guerrero@unison.mx (D.E.G.-M.); juan.galvez@unison.mx (J.C.G.-R.); jhovanna.perez@unison.mx (C.J.P.-M.); judas.vargas@unison.mx (J.T.V.-D.); 2 Department of Medicine and Health Sciences, University of Sonora, Luis Encinas y Rosales, Centro, Hermosillo 83000, Sonora, Mexico; ilokiannette@gmail.com (A.P.I.-L.); sergio.trujillo@unison.mx (S.T.-L.)

**Keywords:** *Terminalia buceras*, *Bucida buceras*, retinal pigment epithelium, ARPE-19, oxidative injury, phytochemical characterization, dry age-related macular degeneration

## Abstract

Dry age-related macular degeneration (dAMD) is a multifactorial retinal disorder involving oxidative stress, mitochondrial dysfunction, chronic para-inflammation, complement dysregulation, and progressive retinal pigment epithelium (RPE) failure. This critical review evaluates the published evidence concerning *Terminalia buceras* (L.) C.Wright (syn. *Bucida buceras* L.; Combretaceae), hereafter referred to as *T. buceras*, and its relevance to dry age-related macular degeneration. The evidence is organized into five categories: species-specific chemical evidence, species-specific retinal evidence, species-specific non-retinal biological evidence, external isolated-compound evidence, and hypothesis-generating comparisons with related plants or experimental systems. The evidence base remains preliminary. Species-specific retinal data are restricted to in vitro observations, and there are currently no epidemiological studies linking *T. buceras* use to AMD outcomes, no validated animal studies demonstrating efficacy in dAMD, and no clinical studies. Moreover, phytochemical standardization, ocular exposure, metabolism, and retinal bioavailability remain insufficiently characterized. Accordingly, this review does not propose *T. buceras* as a preventive or therapeutic intervention and does not claim superiority over better-characterized medicinal plants. Instead, this review critically synthesizes published species-specific chemical and biological findings and evaluates their methodological limitations. The direct retinal evidence supports partial cytoprotection in an ARPE-19 oxidative-injury model, whereas the non-retinal inflammatory findings concern different preparations and experimental systems. Chemical characterization, cellular responses, and contextual mechanistic evidence are, therefore, considered separately. The available findings do not establish a disease-modifying effect on dAMD, clinically achievable retinal exposure, or chronic ocular safety.

## 1. Introduction

Age-related macular degeneration (AMD) is a leading cause of severe visual impairment in older adults. Non-neovascular disease includes early and intermediate stages and late disease with geographic atrophy [1,2]. A population-based synthesis projected an increasing global AMD burden as populations age [3]. Drusen accumulation and progressive RPE and photoreceptor degeneration are central disease features, with oxidative stress, mitochondrial dysfunction, and inflammatory pathways providing biological context [1,4].

Anti-VEGF therapy has improved the management of neovascular AMD [2]. In geographic atrophy secondary to AMD, complement-inhibitor trials demonstrated slower lesion enlargement [5,6,7]. These anatomical outcomes do not establish regeneration of lost RPE or photoreceptors; OAKS and DERBY did not show differences in key secondary visual-function endpoints at 24 months [5].

Oxidative stress and mitochondrial DNA damage provide disease context [8,9]. Impaired autophagy has been reported in cultured RPE from AMD donors [10]. Reviews discuss oxidative stress, inflammation, and RPE degeneration [11,12]. These findings do not demonstrate that a *T. buceras* preparation modifies the corresponding processes.

Studies of individual phytochemicals and defined preparations report protective outcomes in selected experimental retinal-injury models [13,14,15]. These observations provide context and do not establish efficacy of *T. buceras* in dAMD. *Terminalia buceras* (L.) C.Wright (syn. *Bucida buceras* L.; Combretaceae), hereafter referred to as *T. buceras*, is the currently accepted botanical name of the species historically reported as *B. buceras* in much of the phytochemical and pharmacological literature [16]. The selection of *T. buceras* for focused review is not based on presumed phytochemical uniqueness or superiority over other medicinal plants. Phenolic acids, flavonoids, tannins, and terpenoids are widely distributed among medicinal plant species, and the presence of these phytochemical classes alone does not establish therapeutic relevance. *Ginkgo biloba* has been discussed in a review of neurological disorders [17], and *Ginkgo biloba* and *Panax ginseng* extracts were compared in rat stress models [18]. Those non-retinal sources do not establish a comparative advantage of *T. buceras*. Rather, the rationale for examining *T. buceras* is narrower and is based on the existence of a limited but species-specific experimental sequence comprising phytochemical characterization, direct protection of human retinal pigment epithelial ARPE-19 cells against hydrogen peroxide-induced oxidative injury, and anti-inflammatory activity demonstrated in non-retinal experimental models [19,20,21]. Importantly, to the best of our literature search, no epidemiological studies have associated *T. buceras* use or exposure with AMD incidence or progression, and no documented traditional use specifically directed toward retinal disease has been identified. Furthermore, no validated animal model of dAMD or clinical study has demonstrated the efficacy of *T. buceras* or its derived phytochemicals in preventing or treating AMD. Thus, its ethnomedicinal and pharmacological background provides botanical context but should not be interpreted as evidence of ocular efficacy. Accordingly, this review evaluates the conclusions supported by published species-specific findings and the limitations that prevent their extrapolation to retinal mechanisms or clinical benefit.

In this review, non-neovascular AMD encompasses early and intermediate disease and late disease with geographic atrophy (GA); these stages are not considered therapeutically interchangeable. Experimental oxidative injury in RPE cells represents one component of retinal pathology and is not equivalent to clinical dAMD or GA. Accordingly, partial cytoprotection in ARPE-19 cells cannot establish prevention of AMD, modification of GA progression, or restoration of visual function.

The present review builds on three related publications by members of the author group. A review of oxidative stress and inflammation in AMD examined RPE dysfunction, cellular senescence, angiogenesis, genetic susceptibility, and bioinformatics approaches [11]. A polyphenol review discussed dietary polyphenols, selected retinal signaling pathways, and their potential relevance to AMD [22]. The Sonoran Desert review discussed several botanical sources and metabolite classes, redox regulation, Nrf2/NF-κB signaling, and applications across chronic diseases [23]. These publications share conceptual background with the present manuscript, including oxidative stress, inflammation, and phytochemical signaling. The contribution here is a preparation- and source-specific appraisal of *T. buceras* that separates class-level chemical measurements from confirmed individual constituents, direct ARPE-19 observations from non-retinal biological findings, and measured outcomes from mechanisms proposed by analogy [19,20,21]. This appraisal examines whether the evidence supports each step from chemical composition to cellular response and disease relevance; it does not present shared pathway descriptions, the earlier experimental studies, or a species-specific focus alone as new evidence.

## 2. Methods: Literature Search Strategy

This article was designed as a critical narrative review rather than a systematic review or meta-analysis. Literature searches were performed in PubMed/MEDLINE, Scopus, Web of Science Core Collection, and Google Scholar, with the final search updated on 14 August 2026. Search concepts combined terms related to the botanical species, AMD, retinal biology, phytochemistry, and candidate molecular mechanisms. Representative Boolean combinations included (“*Terminalia buceras*” OR “*Bucida buceras*”) AND (phytochemical OR phenolic OR flavonoid OR tannin* OR triterpen* OR antioxidant*); (“*Terminalia buceras*” OR “*Bucida buceras*”) AND (“retinal pigment epithelium” OR ARPE-19 OR retina* OR “oxidative stress”); and (“dry age-related macular degeneration” OR “non-neovascular AMD” OR “geographic atrophy”) AND (Nrf2 OR NF-κB OR NLRP3 OR complement OR mitochondria* OR mitophagy OR phytochemical*). Peer-reviewed original studies, relevant reviews, clinical studies, regulatory documents, and mechanistic studies published in English were considered. Species-specific phytochemical and biological studies involving authenticated *T. buceras* (syn. *B. buceras*) material were prioritized regardless of publication date because of their limited number. Mechanistic studies involving isolated phytochemicals or other plant species were included only to provide biological context and were not considered evidence of activity or chemical occurrence in *T. buceras*. Studies lacking relevance to retinal biology, phytochemical characterization, AMD pathogenesis, or the proposed preclinical framework were excluded.

Records were screened by title and abstract followed by full-text assessment of potentially relevant publications. Because this was a critical narrative review, no PRISMA-based systematic review procedure, meta-analysis, or formal risk-of-bias tool was applied. Evidence was organized into five categories: (i) species-specific chemical evidence; (ii) species-specific retinal evidence; (iii) species-specific non-retinal biological evidence; (iv) external isolated-compound evidence; and (v) hypothesis-generating comparisons with related plants or experimental systems. These categories distinguish source types and do not represent equivalent levels of evidential strength. External studies were used to provide context rather than establish constituent identity or biological activity in *T. buceras.* As a narrative review, this article does not claim exhaustive retrieval of all eligible publications.

## 3. Brief Disease Context and Current Clinical Management

The Beckman Initiative clinical classification distinguishes early AMD, characterized by medium-sized drusen (≥63 and <125 μm) without AMD-related pigmentary abnormalities; intermediate AMD, characterized by large drusen (≥125 μm) and/or AMD-related pigmentary abnormalities; and late AMD, which includes geographic atrophy and neovascular AMD [24]. This review focuses on non-neovascular disease, including geographic atrophy. Retinal pigment epithelial injury is relevant to dry age-related macular degeneration because the RPE supports photoreceptor function and retinal homeostasis [25,26]. Oxidative stress provides a relevant experimental context for assessing cellular injury [8], but the published *T. buceras* study is limited to an oxidative-injury ARPE-19 model [20]. Its findings do not establish effects on the broader molecular processes of AMD, preservation of retinal structure or visual function, or clinical efficacy.

Management of non-neovascular AMD depends on disease stage. The National Eye Institute identifies intermediate AMD in one or both eyes, or late AMD in one eye with a fellow eye at risk, as settings in which AREDS2 supplementation may help; it does not prevent early AMD from progressing to intermediate disease [27]. The original AREDS trial demonstrated reduced progression to advanced AMD in higher-risk participants receiving antioxidants plus zinc [28]. A later post hoc analysis of AREDS and AREDS2 found slower progression of noncentral GA toward the fovea with selected antioxidant or lutein/zeaxanthin assignments, without a significant reduction in area-based GA progression [29]. These outcomes do not establish reversal of established retinal degeneration.

Pegcetacoplan and avacincaptad pegol are approved in the United States for GA secondary to AMD and require intravitreal administration [30,31]. In OAKS and DERBY, pegcetacoplan reduced GA lesion growth at 24 months by 22% and 19%, respectively, with monthly treatment and by 18% and 16% with every-other-month treatment; key secondary visual-function outcomes did not differ from sham [5]. In GATHER2, monthly avacincaptad pegol reduced the square-root-transformed rate of GA growth by approximately 14% over 12 months compared with sham [6]. Separately, the February 2025 IZERVAY prescribing information reports reductions of 18% over 12 months and 14% over 24 months using non-transformed GA growth slopes, with treatment differences of 0.38 and 0.36 mm^2^/year, respectively; the 24-month estimate concerns monthly dosing in both years [31]. The two-year GATHER2 publication reports GA area growth from baseline to year 2 of 4.46 mm^2^ with continued monthly treatment versus 5.18 mm^2^ with sham, a treatment difference of 0.724 mm^2^ (95% CI: 0.133–1.315), representing a 14% reduction [7]. The two-year area changes and annualized growth slopes express the treatment effect on different time scales and should not be treated as interchangeable quantities. These findings indicate slower lesion enlargement rather than restoration of lost retinal tissue. The prescribing information for both agents warns about endophthalmitis, retinal detachment, increased intraocular pressure, and neovascular AMD [30,31]. For pegcetacoplan, the label reports neovascular AMD rates of 12% with monthly treatment, 7% with every-other-month treatment, and 3% with control by month 24; it also warns of retinal vasculitis or vascular occlusion with potentially severe vision loss [30]. By month 12 in GATHER2, macular neovascularization occurred in 15/225 patients (6.7%) receiving avacincaptad pegol and 9/222 patients (4.1%) receiving sham [6].

Over two years, choroidal neovascularization in the study eye occurred in 11.6% of patients across all avacincaptad pegol treatment schedules versus 9.0% of sham-treated patients [7]. These risks and the repeated-injection requirement are relevant to treatment selection and monitoring [30,31].

Therefore, despite the important therapeutic advance represented by complement inhibition, substantial unmet needs remain for interventions capable of providing broader neuroprotection, improved functional outcomes, reduced treatment burden, and favorable long-term safety. The published findings for *T. buceras* do not establish efficacy comparable to these approved treatments or support its use as a clinical alternative.

## 4. *Terminalia buceras* Phytochemistry

*Terminalia buceras* (L.) C. Wright is the accepted name, with *Bucida buceras* L. as a synonym; Kew records its native range from Central Mexico to Colombia (San Andrés Island) and the Caribbean [16]. Species-specific studies provide class-level chemical measurements [19], an oxidative injury ARPE-19 experiment [20], and non-retinal inflammatory observations [21]. The chemical study compared plant parts and extraction solvents and reported the strongest antioxidant assay capacity for acetone extracts of *T. buceras* under its test conditions [19]. These findings do not establish the individual constituents responsible for retinal-cell cytoprotection or clinical ocular efficacy.

The ethanolic preparation was evaluated in oxidatively challenged ARPE-19 cells, with partial cytoprotection and selected assay-specific responses [20]. The separate aqueous preparation study used LPS-stimulated macrophages and carrageenan-induced rat paw edema [21]. These non-retinal results do not establish a shared mechanism or a retinal multitarget pharmacological profile.

Available species-specific phytochemical evidence supports the presence of broad phytochemical classes in *T. buceras*, including phenolic/tannin fractions, flavonoid-related fractions, and triterpene/steroid-type constituents [19]. However, definitive structural identification and quantitative confirmation of many individual metabolites remain insufficient. Consequently, the precise chemical composition of *T. buceras* extracts, the relative abundance of bioactive constituents, and the compounds primarily responsible for the reported biological activities remain incompletely defined.

The chemical characterization of the preparations used in biological experiments limits interpretation of their activity. Class-level phytochemical measurements do not identify the individual constituents responsible for the observed responses or establish their concentrations in the tested preparations. Consequently, the reported biological effects cannot be assigned to specific metabolites, and chemical equivalence among preparations cannot be assumed [19,20,21].

### 4.1. Phytochemical Composition

Species-specific phytochemical screening and colorimetric measurements describe phenolic/tannin and flavonoid-related fractions in *T. buceras.* The study compared different plant parts and extraction solvents; acetone extracts showed the strongest antioxidant capacity under the reported conditions, assessed using DPPH radical scavenging, ferric reducing antioxidant power (FRAP), and nitroblue tetrazolium-based superoxide scavenging [19]. Table 1 summarizes the plant material, extraction conditions, analytical methods, and class-level measurements. The ethanolic preparation used in the retinal-cell experiment and the aqueous preparation used in the inflammatory models are described separately in Section 6.1 [20,21].

The chemistry of related *Terminalia* species provides a more focused basis for selecting analytical candidates. Juang et al. (2004) characterized hydrolysable tannins and related phenolics in *T. chebula* fruits, including chebulagic acid, chebulinic acid, punicalagin, corilagin, and gallic and ellagic acids [32]. These findings support prioritizing tannin-related constituents during chemical profiling of *T. buceras*, alongside other detectable metabolite classes. They provide a rationale for candidate selection rather than confirmation that these compounds occur in the preparation evaluated in retinal cells.

Species-specific screening and colorimetric analyses support phenolic/tannin, flavonoid-related, and triterpene/steroid classes in *T. buceras* [19]. These measurements do not establish the identity or concentration of individual constituents in the preparation tested in ARPE-19 cells, or their contribution to its cytoprotective activity [20]. Table 1 summarizes the available source-specific chemical evidence. Selected isolated-compound studies are discussed in Section 6.2 solely as contextual evidence.

### 4.2. Current Limitations in Phytochemical Characterization

The main chemical limitation is the absence of a sufficiently resolved profile linking constituent identity and abundance to the preparation tested in ARPE-19 cells. Screening and reference-equivalent colorimetric measurements describe classes but cannot assign the biological response to an individual molecule. Chromatographic profiling, appropriate structural confirmation, and quantitative marker measurements would permit comparison of extract batches and support subsequent activity-guided fractionation [19,20,33,34,35].

Preparation differences are also important. The chemical, retinal-cell, and inflammatory studies used different extraction conditions [19,20,21]. Plant organ, geographical origin, harvesting conditions, solvent, and processing should, therefore, be documented when comparing preparations. Establishing reproducible chemical profiles is necessary before evaluating whether individual constituents or interactions among them explain extract-level activity.

## 5. Limits of Mechanistic Attribution

The species-specific chemical study reports phytochemical-class measurements and chemical antioxidant responses [19]. The retinal-cell study reports partial cytoprotection, selected redox-related outcomes, and condition-dependent changes in caspase-3-like activity [20]. These findings do not establish activation of Nrf2/HO-1, inhibition of NF-κB or NLRP3, complement modulation, preservation of mitochondrial function, or enhanced mitophagy by the tested preparation [20]. Non-retinal inflammatory findings concern a separate preparation and experimental context and cannot establish retinal mechanisms [21]. Selected compound-specific findings are discussed only in Section 6.2 and cannot be attributed to the tested *T. buceras* preparation.

## 6. Experimental Evidence

The experimental evidence is considered according to preparation, model, exposure, and measured endpoint. Section 6.1 summarizes the species-specific retinal-cell and non-retinal studies [20,21], with chemical characterization provided in Section 4 [19]. Section 6.2 and Section 6.3 consider external compound studies and related botanical preparations that help interpret candidate selection and model relevance.

### 6.1. Species-Specific Retinal-Cell and Non-Retinal Experimental Evidence

The retinal-cell study evaluated an ethanolic leaf extract of *T. buceras* in ARPE-19 cells exposed to oxidative injury [20]. Approximate visual estimates from Figure 15 of reference [20] indicate that, after 24 h, the viability at 1200 μM H_2_O_2_ was approximately 35–40% of the control without the extract and 50–55% with 1600 μg/mL of extract. At 1600 μM of H_2_O_2_, the corresponding estimates were approximately 20% and 35–40% of the control. These values are approximate readings from the published graph, rather than numerical means reported by the authors. Because cytoprotection was assessed at a single extract concentration, these results do not establish an optimal concentration or a protective concentration–response relationship. The experimental conditions, controls, replication, and statistical treatment are summarized in Table 2.

A separate study evaluated an aqueous leaf extract in carrageenan-induced rat paw edema [21]. Rats received 62.5, 125, or 250 mg/kg intraperitoneally for three consecutive days before challenge. At 5 h after carrageenan injection, the reported edema inhibition was 40.82 ± 10.11%, 49.35 ± 11.35%, and 91.77 ± 0.91%, respectively. The comparator, nimesulide at 4 mg/kg intraperitoneally, produced 51.63 ± 7.21% inhibition; each experimental group contained five rats. The two lower extract doses were not significantly different from nimesulide, whereas the highest dose produced greater inhibition. These findings concern acute peripheral inflammation and a preparation distinct from the ethanolic extract used in ARPE-19 cells.

Interpretation of the retinal-cell findings depends on the differentiation state of the cultures. ARPE-19 cells maintained under standard short-term conditions may exhibit limited pigmentation, incomplete apical–basal polarization and tight-junction maturation, and reduced expression of differentiation markers such as RPE65. These features constrain assessment of visual-cycle function, epithelial transport, and outer blood–retinal barrier physiology. The phenotype is culture-dependent: Ahmado et al. (2011) and Samuel et al. (2017) demonstrated recovery of several differentiated RPE characteristics under appropriate culture conditions [35,36].

Ref. [20] did not document a mature, differentiated phenotype through pigmentation, polarity, barrier measurements, or RPE65 expression. Its viability and biochemical endpoints, therefore, provide evidence of an acute response to oxidative injury, rather than preservation of mature RPE physiological functions. The bolus and GOx protocols also generate different peroxide exposure dynamics, making exposure conditions important when interpreting their outcomes.

### 6.2. Evidence from Isolated Phytochemicals

The compound-specific studies discussed in this section provide contextual evidence from preparations other than the tested *T. buceras* extract. The examples comprise ellagic acid [37], quercetin [13], kaempferol [14], and green tea extract and specific catechins, including epigallocatechin gallate [15]. Each finding applies to the intervention and model evaluated in its source. These examples do not establish a shared mechanism, the chemical occurrence of these compounds in the tested *T. buceras* preparation, or their contribution to its observed activity [19,20]. Xiao et al. investigated ellagic acid in a paraquat-induced intestinal injury model in piglets and reported attenuation of oxidative stress with changes in Nrf2-related signaling [37]. These intestinal findings provide non-retinal context and do not establish retinal protection. Zhu et al. reported improved viability, reduced apoptosis, and changes in Keap1/Nrf2/ARE signaling in retinal pigment epithelial cells exposed to cigarette smoke extract after quercetin treatment [13]. Al Sabaani reported protection against hydrogen peroxide-induced injury in ARPE-19 cells after kaempferol treatment, with findings involving SIRT1 activation and PARP1 inhibition [14]. Yang et al. investigated green tea extract and specific catechin constituents, including epigallocatechin gallate, in sodium iodate-exposed rats [15]. These findings should not be generalized to catechin as an individual compound or interpreted as mechanisms of the tested *T. buceras* preparation. Neither the occurrence of these compounds in that preparation nor their contribution to its activity is established by these external studies.

The contribution of isolated-compound studies to this appraisal is limited by uncertainty about the composition of the retinally tested preparation. The species-specific findings do not identify the individual constituents responsible for its cellular response or establish whether interactions among its components are additive, synergistic, or antagonistic [19,20]. The contextual studies apply to their respective compounds, preparations, and experimental models [13,14,15]; they do not determine the pharmacological profile of the *T. buceras* extract.

### 6.3. Comparative Evidence from Related Botanical Preparations

A relevant comparison is provided by *Triphala*, a formulation containing *T. chebula*, *T. bellirica*, and *Phyllanthus emblica*. Sivasankar et al. [38] evaluated aqueous and alcoholic *Triphala* extracts and the defined compounds chebulagic acid and chebulinic acid in TGFβ1-stimulated ARPE-19 cells. The concentrations associated with an approximately 50% reduction in MMP-2 expression/activity were 100 μg/mL for the aqueous extract, 50 μg/mL for the alcoholic extract, and 100 μM for each compound. Changes in EMT markers, migration, proliferation, and SMAD-3 phosphorylation provided endpoint-specific evidence relevant to proliferative vitreoretinopathy rather than AMD [38]. *Triphala churna* was also evaluated in diabetic rats at 250, 500, and 1000 mg/kg for four weeks. The study assessed electroretinographic responses and retinal histology, together with systemic biochemical measures and lens oxidative-stress and polyol-pathway endpoints [39]. Together, these studies illustrate how chemically defined candidates and tissue-level endpoints can extend an extract-level observation. Because *Triphala* is a multicomponent formulation, its effects cannot be assigned separately to either Terminalia species or transferred to *T. buceras* (Table 3).

**Table 3:** Evidence matrix: current experimental evidence relevant to the retinoprotective potential of *T. buceras* and candidate individual phytochemicals.

*Evidence Level*	*Experimental Model*	*Intervention*	*Principal Molecular Targets*	*Main Findings*	*Translational Relevance*	*Current Limitations*	*References*
*Direct evidence*	ARPE-19 + H_2_O_2_	*T. buceras* extract	Redox-related endpoints and caspase-3-like activity; upstream targets not established	Partial cytoprotection; changes in thiol content, catalase and reducing capacity; condition-dependent reduction in caspase-3-like activity	Preliminary cellular evidence; dAMD efficacy not established	Single cell line; exposure-dependent responses; pathways and active constituents unresolved	[20]
*Direct evidence*	Chemical antioxidant assays	*T. buceras* extract	Free-radical scavenging	Chemical antioxidant measurements and class-level phytochemical results; individual active constituents not identified	Chemical assay evidence; no retinal efficacy established	No retinal validation	[19]
*Contextual non-retinal evidence*	Paraquat-exposed piglets; intestinal injury	Ellagic acid	Nrf2-associated antioxidant responses	Antioxidant responses in the tested intestinal model	Indirect; non-retinal	Contextual evidence; does not establish activity of the tested *T. buceras* preparation	[37]
*Contextual retinal-cell evidence*	Cigarette smoke extract-exposed RPE cells	Quercetin	Changes in Keap1/Nrf2/ARE signaling	Improved viability; reduced apoptosis	Preclinical; in vitro	Contextual evidence; does not establish activity of the tested *T. buceras* preparation	[13]
*Contextual retinal-cell evidence*	H_2_O_2_-exposed ARPE-19 cells	Kaempferol	SIRT1 activation; PARP1 inhibition	Cytoprotection against oxidative injury	Preclinical; in vitro	Contextual evidence; does not establish activity of the tested *T. buceras* preparation	[14]
*Contextual animal-model evidence*	Sodium iodate-exposed rats	Green tea extract and specific catechins, including EGCG	Antioxidant effects evaluated for the tested preparations	Retinal protective outcomes in the tested injury model	Preclinical; animal model	Cannot be generalized to catechin or attributed to *T. buceras*	[15]

This matrix distinguishes species-specific chemical and cellular observations from contextual compound-specific findings. Each result applies to its tested preparation, experimental model, and measured endpoint; the matrix does not establish clinical efficacy or coordinated multitarget activity of *T. buceras*.

## 7. Evidence Gaps and Minimum Requirements for Further Evaluation

The published evidence supports an initial extract-level cytoprotective observation in ARPE-19 cells, complemented by chemical measurements and non-retinal inflammatory findings obtained with separate preparations [19,20,21]. The main unresolved questions are whether the response is reproducible with a chemically standardized extract, whether it extends to differentiated RPE functions, and whether biologically active exposure can be achieved safely in retinal tissue. A minimum interpretable follow-up study would use a botanically authenticated material, a documented extraction procedure, and a reproducible chemical profile with quantitative markers. Cytoprotection should be assessed across an extract concentration range, with matched vehicle and positive controls, extract-alone toxicity testing, independent biological replicates, and a viability endpoint complementary to MTT. Cell-free interference controls would help distinguish genuine cellular effects from extract-related changes in assay readouts. Confirmation in primary or iPSC-derived RPE with documented differentiation, junctional organization, and barrier function would strengthen physiological relevance. Measurements such as photoreceptor outer-segment phagocytosis or polarized transport could determine whether increased survival accompanies preservation of RPE function. A proposed molecular mechanism would require direct pathway measurements and appropriate perturbation experiments. Progression toward an in vivo efficacy claim would additionally require preparation-specific exposure and safety data. Chemical antioxidant capacity and short-term cellular tolerability alone are insufficient grounds for clinical use or direct ocular administration.

## 8. Conclusions

Published studies provide evidence of species-specific chemical and biological observations, including extract-dependent antioxidant activity and partial cytoprotection in an ARPE-19 oxidative-injury model. The non-retinal inflammatory findings extend the biological description of selected preparations but do not establish retinal efficacy. Neither chemical antioxidant potency nor mechanisms demonstrated for other phytochemicals establish that *T. buceras* modifies dAMD. The available evidence does not establish a clinical indication, achievable retinal exposure, or chronic ocular safety. Its current relevance to dAMD is, therefore, limited to preliminary experimental observations whose disease-level significance remains unresolved.

## Figures and Tables

**Table 1:** Source-specific phytochemical evidence reported for authenticated *Terminalia buceras* (syn. *Bucida buceras*) material.

*Plant Part*	*Extraction Solvent*	*Analytical Method*	*Evidence Level*	*Compound/Class Confirmed*	*Standard Confirmation*	*Quantitative Result*	*Original Reference*
Not specified for screening	Hexane	Qualitative phytochemical screening: Carr–Price and Baljet tests	Class-level detection	Carotenes; lactonic groups	No authentic individual-compound standard confirmation	Qualitative detection only; individual compounds not quantified	[19]
Not specified for screening	Ethanol	Qualitative phytochemical screening; ferric chloride, Shinoda, frothing, ninhydrin and related tests	Class-level detection	Phenols/tannins; flavonoids/anthocyanins; lactonic groups; saponins; amines/amino acids	No authentic individual-compound confirmation	Qualitative detection only	[19]
Not specified for screening	Aqueous	Qualitative phytochemical screening	Class-level detection	Phenols/tannins, flavonoids/anthocyanins and other screening-positive secondary-metabolite classes	No authentic individual-compound confirmation	Qualitative detection only	[19]
Leaves	Acetone	AlCl_3_ and 2,4-dinitrophenylhydrazine colorimetric assays	Quantified phytochemical classes	Total flavonoid subclasses	Quantified as reference-standard equivalents; not structural confirmation of individual flavonoids	Flavonol/flavone-related content: 72.075 ± 8.791 μg CE/mg extract; flavanone/flavanonol-related content: 69.897 ± 4.481 μg PNE/mg extract	[19]
Leaves	Acetone	Antioxidant assay associated with phytochemical characterization	Functional chemical evidence	Phenolic/flavonoid-rich extract; individual constituents not identified	Not applicable	Superoxide radical-scavenging activity reported; IC_50_ omitted (see table note)	[19]
Trunk	Acetone	Folin–Ciocalteu and flavonoid colorimetric assays; antioxidant assays	Quantified class-level evidence	Total phenolics and total flavonoids; no individual constituent established	Equivalent-standard quantification only	DPPH activity reported; IC_50_ omitted (see table note); FRAP-reducing power approximately 4928 ± 281 μM AAE/mg extract	[19]
Trunk	Ethanol	Folin–Ciocalteu and flavonoid colorimetric assays	Class-level/quantitative class evidence	Total phenolics and flavonoid subclasses; individual identities unresolved	No authentic individual-compound confirmation	Flavonol/flavone-related content: 19.119 ± 2.018 μg CE/mg extract; flavanone/flavanonol-related content: 93.999 ± 8.614 μg PNE/mg extract	[19]
Trunk	Methanol	Folin–Ciocalteu and flavonoid colorimetric assays	Quantified phytochemical classes	Total phenolics and total flavonoids	Reference-equivalent quantification; no structural confirmation	Flavonol/flavone-related content: 33.976 ± 5.117 μg CE/mg extract; flavanone/flavanonol-related content: 104.269 ± 15.674 μg PNE/mg extract	[19]
Trunk	Aqueous	Folin–Ciocalteu and flavonoid colorimetric assays	Class-level evidence	Total phenolics and flavonoid subclasses	No authentic individual-compound confirmation	Flavonol/flavone-related content: 40.306 ± 2.892 μg CE/mg extract; flavanone/flavanonol-related content: 107.886 ± 14.794 μg PNE/mg extract	[19]
Stem	Acetone	AlCl_3_ and 2,4-dinitrophenylhydrazine colorimetry	Quantified phytochemical class	Total flavonoids, particularly flavanone/flavanonol-related content	Quantified against reference equivalents; individual flavonoids not structurally confirmed	Flavonol/flavone-related content: 49.980 ± 6.171 μg CE/mg extract; flavanone/flavanonol-related content: 237.273 ± 21.254 μg PNE/mg extract	[19]
Stem	Ethanol	Folin–Ciocalteu and flavonoid colorimetric assays	Class-level/quantitative class evidence	Total phenolics and total flavonoids	Reference-equivalent quantification only	Flavonol/flavone-related content: 24.643 ± 2.957 μg CE/mg extract; flavanone/flavanonol-related content: 49.013 ± 3.959 μg PNE/mg extract	[19]
Stem	Methanol	Folin–Ciocalteu and flavonoid colorimetric assays	Quantified phytochemical classes	Total phenolics and flavonoid subclasses	No authentic individual-compound confirmation	Flavonol/flavone-related content: 34.875 ± 3.671 μg CE/mg extract; flavanone/flavanonol-related content: 64.122 ± 5.262 μg PNE/mg extract	[19]
Stem	Aqueous	Folin–Ciocalteu and flavonoid colorimetric assays	Class-level/quantitative class evidence	Chemically complex aqueous *T. buceras* extract	No authentic individual-compound confirmation	Flavonol/flavone-related content: 41.430 ± 2.510 μg CE/mg extract; flavanone/flavanonol-related content: 62.979 ± 5.292 μg PNE/mg extract	[19]
Leaves	Aqueous extract	Species-specific biological evidence, not individual-compound confirmation	Species-specific biological evidence, not individual-compound confirmation	Chemically complex aqueous *T. buceras* extract	No individual constituent confirmed as responsible for activity	Extract concentrations of 50–400 μg/mL in RAW 264.7 experiments; 62.5–250 mg/kg in the rat model	[21]
*Leaves*	Ethanolic extract	Biochemical antioxidant characterization associated with ARPE-19 experiments	Species-specific retinal biological evidence, not individual-compound confirmation	Complex ethanolic leaf preparation; individual active constituents unresolved	No authentic-standard confirmation linking an individual molecule to retinal protection	Extract tested up to 1600 μg/mL in ARPE-19 cells; cytoprotective effects assessed under H_2_O_2_/GOx oxidative stress	[20]

**Abbreviations:** CE, catechin equivalents; PNE, pinocembrin equivalents; AAE, ascorbic acid equivalents. Equivalent-based measurements describe assay responses relative to calibration standards and do not establish the presence of those compounds in the extract. Only species-specific evidence directly obtained using *Terminalia buceras* (syn. *Bucida buceras*) material is shown. “Class-level detection” indicates qualitative phytochemical screening or spectrophotometric quantification and should not be interpreted as definitive structural identification of individual metabolites. Reference-equivalent measurements such as total phenolic or flavonoid content provide quantitative class-level information but do not establish the presence of specific compounds such as quercetin, kaempferol, rutin, luteolin, catechin, lupeol, α-amyrin, or β-amyrin. Where individual-compound identity was not confirmed using appropriate chromatographic/spectrometric methods and authentic standards, the compound should not be presented as an established constituent of *T. buceras*. Refs. [20,21] are included only to document the species-specific extracts used in retinal and anti-inflammatory biological studies; they do not constitute independent confirmation of specific individual phytochemicals. Chemical antioxidant measurements depend on the assay system, reaction conditions, concentration range, and extract preparation tested. Standards reported in other publications are not matched experimental controls. Standalone IC_50_ values are, therefore, omitted from this table; no unavailable comparator measurements have been inferred. These functional assays do not establish individual-compound identity or retinal efficacy. PNE, pinocembrin equivalents. Values expressed as μg PNE/mg extract represent colorimetric calibration equivalents and do not establish the presence or concentration of pinocembrin itself in the plant material [19].

**Table 2:** Experimental conditions and reported outcomes of the species-specific retinal-cell study [20].

*Parameter*	*Reported Information*
Extract-alone concentrations	12.5–1600 μg/mL in the text; the inset of Figure 15 of reference [20] specifies 50–1600 μg/mL.
Protective treatment	1600 μg/mL; bolus H_2_O_2_; 24 h.
Bolus H_2_O_2_, 1200 μM; 24 h	Viability: approximately 35–40% without extract versus 50–55% with 1600 μg/mL extract.
Bolus H_2_O_2_, 1600 μM; 24 h	Viability: approximately 20% without extract versus 35–40% with 1600 μg/mL extract.
GOx-generated peroxide	GOx at 12.5, 25, and 50 mU/mL was used in biochemical co-treatment experiments. A paired MTT viability comparison with versus without extract was not identifiable in the published results.
Vehicle control	0.1% DMSO control reported.
Protective concentration–response	Not demonstrated; cytoprotection was evaluated at 1600 μg/mL extract.
Positive comparator	Catalase, 200 U/mL, in selected GOx endpoint experiments; not a matched comparator in Figure 15 of reference [20].
Extract-alone toxicity	Low overall; minor cytotoxicity reported at 1600 μg/mL.
Replication	Figure 15 of reference [20]: 5–7 independent experiments.
Statistical analysis	Mean ± SD; GLM-ANOVA followed by Tukey testing; *p* ≤ 0.05.

Bolus viability values are approximate visual estimates from Figure 15 of reference [20], expressed relative to control cells (100%). GOx-related biochemical outcomes are distinguished from MTT viability measurements. Table footnote: DMSO, dimethyl sulfoxide; GOx, glucose oxidase; SD, standard deviation.

## Data Availability

No new data were created or analyzed in this study. Data sharing is not applicable to this manuscript.

## Abbreviations

AAE	Ascorbic acid equivalents
AI	Artificial intelligence
AMD	Age-related macular degeneration
AREDS	Age-related eye disease study
CE	Catechin equivalents
dAMD	Dry age-related macular degeneration
DMSO	dimethyl sulfoxide
DNA	Deoxyribonucleic acid
DPPH	2,2-diphenyl-1-picrylhydrazyl
FDA	US Food and Drug Administration
FRAP	Ferric-reducing antioxidant power
GA	Geographic atrophy
GAE	Gallic acid equivalents
GOx	Glucose oxidase
H_2_O_2_	Hydrogen peroxide
HO-1	Heme oxygenase 1
iPSC	Induced pluripotent stem cell
MTT	3-(4,5-dimethylthiazol-2-yl)-2,5-diphenyltetrazolium bromide
NF-κB	Nuclear factor kappa B
NLRP3	NOD-, LRR-, and pyrin domain-containing protein 3
Nrf2	Nuclear factor erythroid 2-related factor 2
PNE	Pinocembrin equivalents
RPE	Retinal pigment epithelium
SD	Standard deviation
SIRT1	Sirtuin 1
VEGF	Vascular endothelial growth factor
